# Inflammatory markers associated with albuminuria and early atherosclerosis in type 2 diabetic kidney disease: a cross-sectional study

**DOI:** 10.3389/fendo.2026.1732900

**Published:** 2026-02-26

**Authors:** Ainhoa González-Luis, Orlando Siverio-Morales, Carolina Hernández-Carballo, Carmen Mora-Fernández, Ernesto Martín-Núñez, Andrea Reyes-Carrión, J. Diego Carlos-Monzón, Juan F. Navarro-González, Javier Donate-Correa

**Affiliations:** 1 Unidad de Investigación, Hospital Universitario Nuestra Señora de Candelaria, Santa Cruz de Tenerife, Spain; 2 Instituto de Tecnologías Biomédicas, Universidad de La Laguna, Santa Cruz de Tenerife, Spain; 3 Doctoral and Graduate School, University of La Laguna, San Cristóbal de La Laguna, Spain; 4 Nephrology Service, Hospital Universitario Nuestra Señora de Candelaria, Santa Cruz de Tenerife, Spain; 5 Internal Medicine Service, Hospital Universitario Nuestra Señora de Candelaria, Santa Cruz de Tenerife, Spain; 6 GEENDIAB (Grupo Español para el Estudio de la Nefropatía Diabética), Sociedad Española de Nefrología, Santander, Spain; 7 RICORS2040 (RD24/0004/0022), Instituto de Salud Carlos III, Madrid, Spain; 8 Cardiovascular Translational Research. Navarrabiomed (Fundación Miguel Servet), Instituto de Investigación Sanitaria de Navarra (IdiSNA), Hospital Universitario de Navarra (HUN), Universidad Pública de Navarra (UPNA), Pamplona, Spain; 9 Facultad de Ciencias de la Salud, Universidad Fernando Pessoa Canarias, Las Palmas de Gran Canaria, Spain

**Keywords:** albuminuria, atherosclerosis, chronic kidney disease, inflammation, type 2 diabetes

## Abstract

**Background:**

Albuminuria is a recognized marker of renal injury in diabetic kidney disease (DKD), and growing evidence suggests it may also drive systemic inflammation and atherosclerosis. However, mechanisms linking albuminuria to vascular disease remain unclear.

**Methods:**

We conducted a cross-sectional study including 362 subjects with type 2 diabetes and moderate chronic kidney disease (CKD) to evaluate the associations between albuminuria, circulating and leukocyte inflammatory markers, and measures of subclinical atherosclerosis (SA). SA was defined by carotid intima-media thickness (CIMT) ≥0.9 mm or ankle-brachial index (ABI) <0.9. Serum levels of hs-CRP, IL6, IL1β, IL10, and TNFα and gene expression in peripheral blood leukocyte cells for *IL6, IL1β, TNF, IL10, TLR2, TLR4, CCL2, NFκB*, and *CD36* were measured.

**Results:**

SA was present in 46% of patients. UACR correlated directly with CIMT (r=0.32, p<0.001) and inversely with ABI (r=–0.29, p<0.01). Higher UACR was associated with increased circulating IL6 and IL1β, and elevated expression of *TNF*, *CD36*, and *TLR2* in leukocytes. In multivariable analysis, serum IL6and IL1β, and leukocyte *IL6* and *TLR2* were independently associated with SA. Mediation analysis showed that IL6 (serum and leukocyte expression) accounted for approximately 20% of the UACR–SA association, serum IL1β mediated 17%, and leukocyte *TLR2* mediated 7%.

**Conclusions:**

Albuminuria was positively associated with heightened systemic and cellular inflammation, and several inflammatory markers were also associated with greater CIMT and the presence of early atherosclerosis. Exploratory mediation analyses suggested that inflammatory pathways may partly account for the association between albuminuria and SA, with IL6, IL1β, and TLR2 as key mediators; however, these findings should be interpreted cautiously due to the cross-sectional design.

## Introduction

Albuminuria is a common manifestation of diabetic kidney disease (DKD), a major complication of diabetes mellitus, and has been recognized as a marker not only of renal injury but also of systemic vascular pathology, including subclinical atherosclerosis (SA) (1–3). Large epidemiologic studies have further established chronic kidney disease (CKD) markers as risk factors for cardiovascular events; thus, both reduced glomerular filtration and elevated urinary albumin-to-creatinine ratio (UACR) confer significantly higher risks of cardiovascular morbidity and mortality (4, 5). Even low-grade albuminuria (10–30 mg/g) has been independently associated with early indicators of SA, such as increased carotid intima-media thickness (CIMT) and abnormal ankle-brachial index (ABI) (6–9). However, the mechanistic pathways linking albuminuria to vascular injury remain only partially elucidated.

Chronic low-grade inflammation is a well-established contributor to both DKD progression and atherogenesis (10–13). In type 2 diabetes, chronic low-grade inflammation arises from multiple metabolically active tissues. Expansion of visceral adipose tissue and the frequent coexistence of non-alcoholic fatty liver disease (NAFLD) contribute to systemic cytokine release and have been linked to early atherosclerotic changes, including increased carotid intima-media thickness (14). These metabolic inflammatory sources may interact with kidney-associated inflammatory pathways in diabetic kidney disease, amplifying vascular risk.

A growing body of evidence suggests that albuminuria may not merely reflect renal endothelial dysfunction but may actively amplify systemic inflammation through tubular cell activation, release of pro-inflammatory mediators, and recruitment of immune cells. Inflammatory cytokines such as tumor necrosis factor α (TNFα), interleukin (IL) IL6, and IL1β are upregulated in patients with renal impairment and directly contribute to vascular remodeling and plaque instability (15, 16). Circulating peripheral blood leukocytes represent a key source of these inflammatory mediators and have been implicated in the development of atherosclerotic lesions as well as the abnormal immune activation observed in CKD (17). Notably, pro-inflammatory monocyte subsets (CD14^+^CD16^+^) are expanded in T2DM patients with nephropathy and are associated with microinflammation in DKD, highlighting the link between systemic immune cell activation and renal injury (18).

Alterations in blood leukocyte RNA, particularly those involving inflammatory cytokines, pattern recognition receptors (e.g., toll-like receptor [TLR]), chemokines (e.g., C-C motif chemokine ligand 2 [CCL2]), and transcription factors (e.g., nuclear factor kappa B [NFκB]), have been reported in both DKD and cardiovascular disease (19–21). Nevertheless, few studies have simultaneously assessed the interplay between UACR, systemic inflammatory mediators, circulating leukocyte gene expression, and SA in a well-characterized population with DKD. This represents a critical knowledge gap in understanding the kidney–heart axis.

In this cross-sectional study, we investigated the hypothesis that albuminuria serves, at least in part, as a biological mediator linking systemic inflammation to early atherosclerosis in DKD. We analyzed a cohort of patients with T2DM and moderate CKD (stages G3a/b) to explore the associations between UACR, circulating inflammatory parameters (high-sensitivity C-reactive protein [hs-CRP], TNFα, IL6, IL1β, IL10), and gene expression profiles in peripheral blood leukocytes (including *TNF*, *IL6*, *IL1B*, *IL10*, *CD36*, *CCL2*, *TLR2*, *TLR4*, and *NFKB*). Vascular outcomes were evaluated using CIMT and ABI as markers of subclinical arterial damage. Our findings offer novel insights into the role of albuminuria as an active participant in the inflammatory-atherosclerotic axis in DKD, providing context for potential therapeutic interventions targeting inflammation.

## Methods

### Study design and participants

This cross-sectional, single-center study was conducted at the University Hospital Nuestra Señora de Candelaria (Santa Cruz de Tenerife, Spain). Participants were selected from patients with type 2 diabetes mellitus (T2DM) with no history of clinical atherosclerotic cardiovascular disease (CVD) who had a clinical indication for SA evaluation, assessed by measuring the ankle-brachial index (ABI) and carotid intima-media thickness (CIMT) between May and November 2010. During this study period, SGLT2 inhibitors and finerenone were not yet available or used in clinical practice; therefore, none of the participants were receiving these albuminuria-lowering agents.

Inclusion criteria were: diagnosis of T2DM for more than 1 year; CKD stage G3a/b (estimated glomerular filtration rate [eGFR] 30–59 mL/min/1.73 m² according to the Modification of Diet in Renal Disease Study-4 [MDRD-4] equation); UACR ≥30 mg/g; age over 18 years; and stable treatment with renin-angiotensin system (RAS) blockers and statins. CKD status and albuminuria were determined according to KDIGO criteria, based on repeated eGFR and UACR measurements obtained over ≥3 months. Only patients with persistent UACR ≥30 mg/g were eligible for inclusion.

Exclusion criteria included: ABI values ≥1.3 (indicating non-compressible arteries); history of heart failure; active malignancy or chronic inflammatory, immunologic, or infectious disease; positive serology for hepatitis B, C, or HIV; acute illness within one month; institutionalization; use of immunotherapy or immunosuppressants; or inability to provide informed consent. Similarly, Patients with recent exposure to non-steroidal anti-inflammatory drugs or short courses of antibiotics, or other nephrotoxic agents known to acutely affect UACR or renal function, were excluded or required to have repeat measurements confirming UACR stability. The study was approved by the local ethics committee, and all participants gave written informed consent.

### Subclinical atherosclerosis assessment

SA was defined as the presence of either and ABI <0.9 and/or CIMT ≥0.9 mm, according to the guidelines of the Task Force for the Management of Arterial Hypertension of the European Society of Hypertension (ESH) and the European Society of Cardiology (ESC) (22). ABI was measured in supine position using a Doppler ultrasound device (Ultrasonic Mini Doppler ES-100VX; Hayashi Denki Co., Ltd., Kawasaki, Japan) equipped with an 8 MHz probe. The lowest ankle-to-brachial systolic pressure ratio of either leg was used. CIMT was measured by high-resolution B-mode ultrasonography (Philips ATL 5000 HDI; Royal Philips Electronics, Amsterdam, The Netherlands) with a 6–13 MHz linear array transducer. A single experienced reader, blinded to clinical data, measured CIMT in distal common carotid arteries; the mean of near- and far-wall measurements (1 cm proximal to the bifurcation) on both sides was taken as representative CIMT.

### Biochemical and inflammatory marker measurements

Blood samples were obtained in the morning following an overnight fast of 8 to 12 hours using PAXgene Blood RNA tubes (BD, Franklin Lakes, NJ) and standard blood collection tubes (BD Serum Separation Transport Tubes; BD, Franklin Lakes, NJ). Routine biochemical parameters were measured by standard automated laboratory methods. Circulating TNFα, IL1β, IL6, and IL10 were quantified by high-sensitivity ELISA kits (Quantikine^®^, R&D Systems, Abingdon, UK) following the protocols of the manufacturer. The assay detection limits were 0.10 pg/mL for TNF-α/IL-1β and 0.09 pg/mL for IL-6/IL-10; intra- and inter-assay coefficients of variation were <10.8%. Serum hs-CRP by a particle-enhanced immunoturbidimetric assay (Roche Diagnostics, Mannheim, Germany) on a Cobas 6000 analyzer (analytical sensitivity 0. 3mg/L; intra-/inter-assay CV 1.6%/8.4%). In addition, two composite indices related to systemic inflammation were calculated: the neutrophil-to-lymphocyte ratio (NLR) and the systemic immune-inflammation index (SII) defined as platelet count x neutrophil count/lymphocyte count).

### Peripheral blood leukocyte gene expression analysis

Whole blood was collected into PAXgene Blood RNA tubes (PreAnalytiX/QIAGEN, Hilden, Germany) for immediate stabilization of intracellular RNA. After storage and processing according to the instructions of the manufacturer, total RNA was extracted from the stabilized blood leukocyte fraction. Thus, gene expression measurements reflect circulating peripheral blood leukocytes, which include a predominance of mononuclear cells. To avoid ambiguity, we refer to these measurements as peripheral blood leukocyte gene expression, acknowledging that they predominantly reflect mononuclear cells within the whole-blood leukocyte population. RNA quantity and purity were verified by spectrophotometry, and 1 μg of RNA was reverse-transcribed to cDNA using a High-Capacity RNA-to-cDNA kit (Thermo Fisher Scientific, Waltham, MA, USA). Quantitative real-time PCR (qRT-PCR) was performed on an ABI 7500 Fast instrument (Applied Biosystems) using TaqMan gene expression assays for target genes: *TNF*, *IL1β*, *IL6*, *IL10*, *CD36*, *CCL2*, *NFκB1* (NFκB p50 subunit), *TLR2* and *TLR4* (detailed in **Supplementary Table S1**). Expression of each target was normalized to the housekeeping gene *GAPDH*. Relative mRNA expression was calculated using the 2^−ΔΔCt^ method, with results expressed in arbitrary units. All qRT-PCR reactions were run in triplicate, and technicians were blinded to clinical data.

### Statistical analysis

Continuous variables were assessed for normality using the Kolmogorov–Smirnov test. Variables with a normal distribution are presented as mean ± standard deviation (SD), while non-normally distributed data are reported as median and interquartile range (IQR). Skewed variables (including UACR, cytokine levels, and gene expression values) were log-transformed for parametric analyses. Categorical variables were presented as counts and percentages. Group comparisons were performed using the Chi-square test, Student’s t-test, or the Mann–Whitney U-test, as appropriate. Spearman’s rank correlation coefficient was used to evaluate associations between continuous variables.

We employed multivariable logistic regression models to examine the associations between inflammatory markers and the presence of SA. Potential vascular confounders—including age, sex, BMI, hypertension, smoking status, and lipid profile parameters—were evaluated; the final multivariable models only included those that contributed significantly without introducing collinearity. For this purpose, three incremental models were constructed: model 1 (unadjusted); model 2 (adjusted for age, sex, and body mass index -BMI-); and model 3 (adjusted for age, sex, BMI, UACR, eGFR, smoking status, and hypertension). The results were expressed as odds ratios (ORs) with 95% confidence intervals (CI). We then preformed mediation analysis (using a bootstrap approach with 5,000 resamples) to quantify the extent to which inflammatory markers mediated the association between UACR and SA. For each mediator, we estimated the total, direct, and indirect effects of UACR on SA, and calculated the proportion mediated along with 95% CIs. A two-sided P value of < 0.05 was considered statistically significant. To account for multiple testing, we applied the Benjamini–Hochberg false discovery rate (FDR) correction within pre-specified test families: (1) serum inflammatory biomarkers vs vascular outcomes (ABI, CIMT, SA); (2) leukocyte gene expression markers vs vascular outcomes; and (3) mediation models evaluating inflammatory markers as mediators between UACR and SA. All statistical analyses were performed using SPSS Statistics software, version 25.0 (IBM Corp. Armonk, NY, USA). Mediation analyses were conducted using the PROCESS macro (version 3.5) developed by Andrew F. Hayes (22), applying a non-parametric bootstrap procedure with 5,000 resamples to estimate indirect effects and their 95% confidence intervals. In mediation models, UACR was specified as the exposure variable rather than as a generic covariate, with covariate adjustment applied to the exposure–mediator–outcome paths as appropriate (23).

## Results

### Participant characteristics

**Figure 1:**
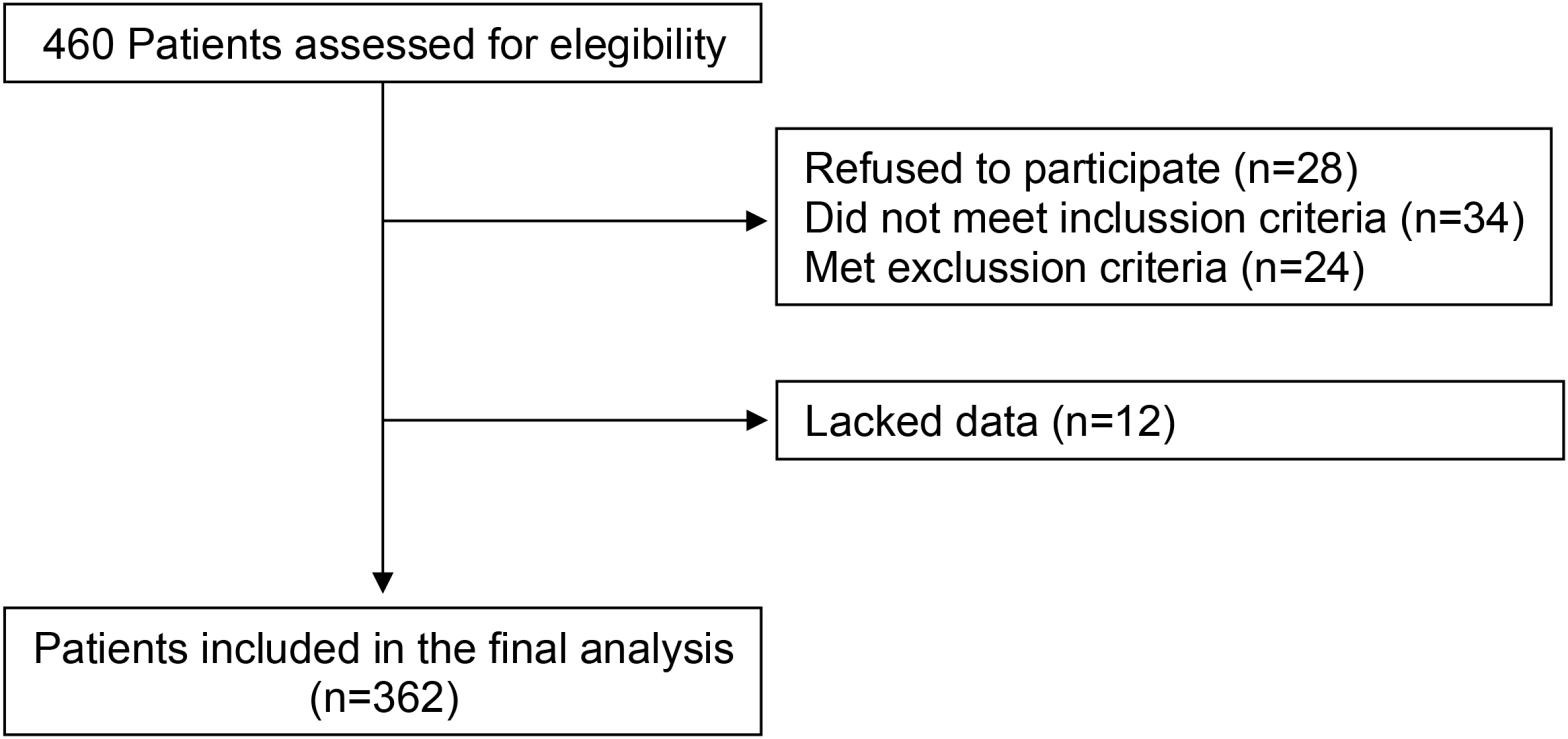
Flowchart detailing the process of patient selection.

**Figure 1** displays the flowchart of participant selection throughout the study. A total of 460 patients were initially screened, of whom 98 were excluded (28 declined participation, 34 did not meet inclusion criteria, 24 met at least one exclusion criterion, and had incomplete data). The final analysis included 362 patients with T2DM and moderate CKD. The mean age was 67.3 ± 7.9 years, 49.2% were male, and by design none one had a history of clinical cardiovascular disease. The median eGFR was 38.6 [35.5-43.9] mL/min/1.73 m². All patients were receiving treatment with RAS blockers and statins.

**Table 1** summarizes the demographic, clinical, and biochemical characteristics of the entire study population, as well as according to the presence of SA. SA was present in 165 patients (45.6%), of whom 135 had an ABI < 0.9 and 75 had a CIMT ≥ 0.9 mm. Compared to patients without SA, patients with SA had significantly higher CIMT values (0.79 [0.74–0.89] vs. 0.72 [0.69–0.78] mm, p < 0.001) and lower ABI values (0.855 ± 0.05 vs. 1.04 ± 0.09, p < 0.001). No significant differences in age, sex distribution, blood pressure, BMI, glycemic control, or lipid profile were observed between groups. Similarly, medication use, including insulin, oral antidiabetic agents, and antiplatelet therapy, was comparable across groups. However, the prevalence of smoking was significantly higher in patients with SA (31.7% vs. 24.9%, p = 0.02). By contrast, UACR levels were significantly elevated in patients with SA (979 [346–1302] vs. 219 [133–594] mg/g, p < 0.001) (**Figure 1**). Additionally, a higher proportion of patients with SA had macroalbuminuria (78.8% vs. 35.5%, p < 0.001).

**Table 1:** Demographic, clinical and biochemical baseline characteristics of patients according to the presence of subclinical atherosclerosis.

Variable	All subjects	SA	No SA	*p*
Characteristics				
N	362	165	197	
Age (years)	67.3 ± 7.9	67.9 ± 8.1	66.9 ± 7.8	0.543
Male (%)	178 (49.2)	86 (52.1)	92 (46.7)	0.357
Systolic BP (mm Hg)	140 (136-150)	140 (134-148)	140 (138-151)	0.511
Diastolic BP (mm Hg)	88 (82-90)	89 (82-90)	86 (82-90)	0.512
BMI (kg/m^2^)	28.9 ± 2.9	29 ± 3.5	28.9 ± 2.7	0.938
ABI	0.967 ± 0.94	0.855 ± 0.05	1.04 ± 0.09	<0.001
CIMT (mm)	0.75 (0.7-0.82)	0.79 (0.74-0.89)	0.72(0.69-0.78)	<0.001
Comorbidities (%)				
Hypertension	315 (87)	148 (89.7)	167 (84.8)	0.282
Smokers	124 (34.3)	71 (43)	53 (26.9)	0.02
Dyslipidemia	270 (74.6)	118 (71.5)	152 (77.2)	0.355
Macroalbuminuria	200 (55.2)	130 (78.8)	70 (35.5)	<0.001
Laboratory values				
Hemoglobin (g/dl)	11.8 (11.4-12.1)	11.9 (11.5-12.4)	11.8 (11.3-12.1)	0.3
HbA1c (%)	7.21 ± 0.68	7.16 ± 0.57	7.27 ± 0.81	0.623
Creatinine (mg/dl)	1.9 (1.48-2.62)	1.87 (1.55-2.39)	1.9 (1.38-2.79)	0.651
eGFR (ml/min/1.73 m^2^)	38.6 (35.5-43.9)	38.6 (34.4-43.3)	38.6 (36.1-46.7)	0.339
UACR (mg/g)	374 (174-1033)	979 (346-1302)	219 (133-594)	<0.001
Albumin (g/dL)	3.77 ± 0.65	3.75 ± 0.37	3.79 ± 0.79	0.718
T-cholesterol (mg/dL)	172.3 ± 36.3	173.4 ± 37.7	171.5 ± 35.6	0.803
HDL-C (mg/dL)	42.3 ± 10.2	43.6 ± 10	41.3 ± 10.3	0.255
LDL-C (mg/dL)	98 (80-113)	106 (79-116)	91.5 (80-108.8)	0.18
Triglycerides (mg/dL)	150.7 ± 40.9	143.7 ± 42.6	155.5 ± 39.4	0.159
Glucose (mg/dL)	107.4 ± 36	114.5 ± 36.3	102.4 ± 35.2	0.095
Neutrophils (x10^9^/L)	5.6 ± 2.3	5.4 ± 1.3	5.7 ± 1.9	0.561
Lymphocytes (x10^9^/L)	2.2 ± 1.2	1.9 ± 1.1	2.3 ± 1.2	0.723
Platelets (x10^9^/L)	209 ± 76.1	211 ± 82	207 ± 65	0.536
Uric acid (mg/dL)	6.62 ± 1.47	6.59 ± 1.3	6.64 ± 1.59	0.882
Calcium (mg/dL)	9.3 ± 0.57	9.3 ± 0.53	9.3 ± 0.59	0.676
Phosphorus (mg/dL)	4.37 ± 0.69	4.33 ± 0.67	4.39 ± 0.72	0.629
Medication (%)				
Insulin	103 (28.5)	48 (29.1)	55 (27.9)	0.321
Oral antidiabetics	209 (57.7)	90 (54.5)	119 (60.4)	0.254
Antiaggregant	50 (13.8)	20 (12.1)	30 (15.2)	0.272

BMI, body mass index; ABI, ankle-brachial index; CIMT, carotid intima-media thickness; HbA1ac, glycated hemoglobin; eGFR, estimated glomerular filtrate rate; UACR, urine albumin-to-creatinine ratio; T-cholesterol, total cholesterol; LDL-C low-density lipoprotein cholesterol; HDL-C high density lipoprotein cholesterol.

### Inflammatory parameters by albuminuria and SA status

Serum concentrations, composite inflammatory indices, and blood leukocyte mRNA expression levels of inflammatory markers were compared between groups stratified by the presence of SA and the degree of albuminuria (**Table 2**). After applying the Benjamini–Hochberg FDR correction, only a subset of associations remained statistically significant, whereas others became attenuated. Patients with macroalbuminuria exhibited significantly higher serum levels of hs-CRP compared to those with lower albuminuria (5.76 ± 2.41 *vs.* 4.73 ± 2.29 mg/L, *p* = 0.029). Similarly, circulating concentrations of IL6 and IL1β were markedly elevated both in the macroalbuminuric group (IL6: 6.1 [3.4–8.5] vs. 3.1 [2.4–5.8] pg/mL, *p* < 0.001; IL1β: 0.51 [0.21–0.79] *vs.* 0.42 [0.22–0.79] pg/mL, *p* < 0.01) and in patients with SA (IL6: 6.8 [3.8–9.52] *vs.* 3.3 [2.4–5.7] pg/mL, *p* < 0.001; IL1β: 0.45 [0.19–0.87] *vs.* 0.37 [0.11–0.78] pg/mL, *p* < 0.01). Among the composite indices of systemic inflammation, only the SII differed significantly between groups, with higher values observed in patients with macroalbuminuria (602 [301–952] *vs*. 571 [310–899], *p* = 0.02).

**Table 2:** Inflammation-related parameters according to the presence of microalbuminuria or subclinical atherosclerosis.

Variable	SA (N = 165)	No SA (N = 197)	*p*	Macroalbuminuria (N = 200)	Microalbuminuria (N = 46)	*p*
Circulating levels						
hs-CRP (mg/L)	5.34 ± 2.5	5.26 ± 2.36	0.852	5.76 ± 2.41	4.73 ± 2.29	0.029
TNFα (pg/mL)	14.9 (11.7-17.7)	15.6 (12.1-17.9)	0.833	15.5 (11.1-17.6)	15.6 (12.7-18.5)	0.425
IL6 (pg/mL)	6.8 (3.8-9.52)	3.3 (2.4-5.7)	<0.001	6.1 (3.4-8.5)	3.1 (2.4-5.8)	<0.001
IL1β (pg/mL)	0.45 (0.19-0.87)	0.37 (0.11-0.78)	<0.01	0.51 (0.21-0.79)	0.42 (0.22-0.79)	<0.01
IL10 (pg/mL)	35 (29.4-43.3)	36.8 (24.2-49.1)	0.992	36.4 (29.5-44.7)	33.7 (22.9-53.6)	0.643
Indices for systemic inflammation						
SII (x10^9^/L)	595 (292–981)	560 (210-1010)	0.021	602 (301–952)	571 (310-899)	0.02
NLR	2.51 (1.8-3.2)	2.46 (1.59-3.1)	0.254	2.53 (1.8-3.3)	2.47 (1.75-3.1)	0.301
Blood leukocytemRNA (a.u.)						
*TNF*	1.95 (1.61-2.78)	1.75 (1.24-2.3)	0.031	1.95 (1.7-2.7)	1.8 (1.4-2.4)	0.04
*IL1β*	1.36 (0.81–2.67)	1.21 (0.73–2.31)	0.893	1.52 (0.72–2.71)	1.17 (0.83–2.10)	0.03
*IL6*	1.8 (1.5-2.4)	1.8 (1.53-2.2)	0.929	1.8 (1.4-2.4)	1.85 (1.5-2.2)	0.893
*IL10*	1.27 (1.08-1.4)	1.25 (1.12-1.37)	0.948	1.28 (1.11-2.3)	1.26 (1.1-1.4)	0.677
*CD36*	1.6 (1.1–2.43)	1.2 (0.9–2.11)	0.01	1.8 (1.29–2.7)	1.3 (0.87–1.8)	<0.01
*CCL2*	0.37 (0.29–0.61)	0.31 (0.17–0.57)	0.542	0.37 (0.29–0.61)	0.31 (0.17–0.57)	0.542
*TLR2*	1.21 (0.54–4.17)	1.03 (0.61–2.82)	0.013	1.37 (0.44–3.97)	0.85 (0.42–2.95)	0.021
*TLR4*	1.29 (0.81–2.62)	1.19 (0.78–2.22)	0.039	1.35 (0.77–2.86)	1.03 (0.85–2.37)	0.041
*NFkB*	1.2 (0.79–2.32)	1.21 (0.82–2.91)	0.601	1.22 (0.87–2.22)	1.14 (0.79–2.68)	0.571

hs-CRP, high sensitivity C-reactive protein, TNFα, tumor necrosis factor alpha; IL, interleukin; SII, systemic immune-inflammation index; NLR, neutrophil-to-lymphocyte ratio; CD36, cluster differentiation 36; CCL2, C-C motif chemokine ligand 2; TLR, toll-like receptor; NFκB, nuclear factor kappa B.

The mRNA expression levels of *TNF*, *CD36*, *TLR2* and *TLR4* differed significantly between patients with and without SA, showing higher values in those with SA (TNF: 1.95 [1.61–2.78] *vs.* 1.75 [1.24–2.3] a.u.; CD36: 1.6 [1.1–2.43] *vs*. 1.2 [0.9–2.11] a.u.; *TLR2:* 1.21 [0.54–4.17] vs. 1.03 (0.61–2.82) a.u.; *TLR4:* 1.29 [0.81–2.62] vs. 1.19 [0.78–2.22] a.u.; p < 0.05 for all). Likewise, patients with macroalbuminuria also exhibited elevated mRNA expression levels of all these parameters when compared to those with microalbuminuria (TNF: 1.95 [1.7–2.7] *vs.* 1.8 [1.4–2.4] a.u., *p* = 0.04; CD36 (1.8 [1.29–2.7] *vs*. 1.3 [0.87–1.8] a.u., *p* < 0.01; *TLR2:* 1.37 [0.44–3.97] vs. 0.85 (0.42–2.95) a.u., p = 0.021; *TLR4:* 1.35 [0.77–2.86] vs. 1.03 [0.85–2.37] a.u., p = 0.041). Furthermore, the macroalbuminuric group also exhibited increased mRNA expression of IL1β (1.52 [0.72–2.71] *vs*. 1.17 [0.83–2.10] a.u., *p* = 0.03).

### Correlations

**Figure 2:**
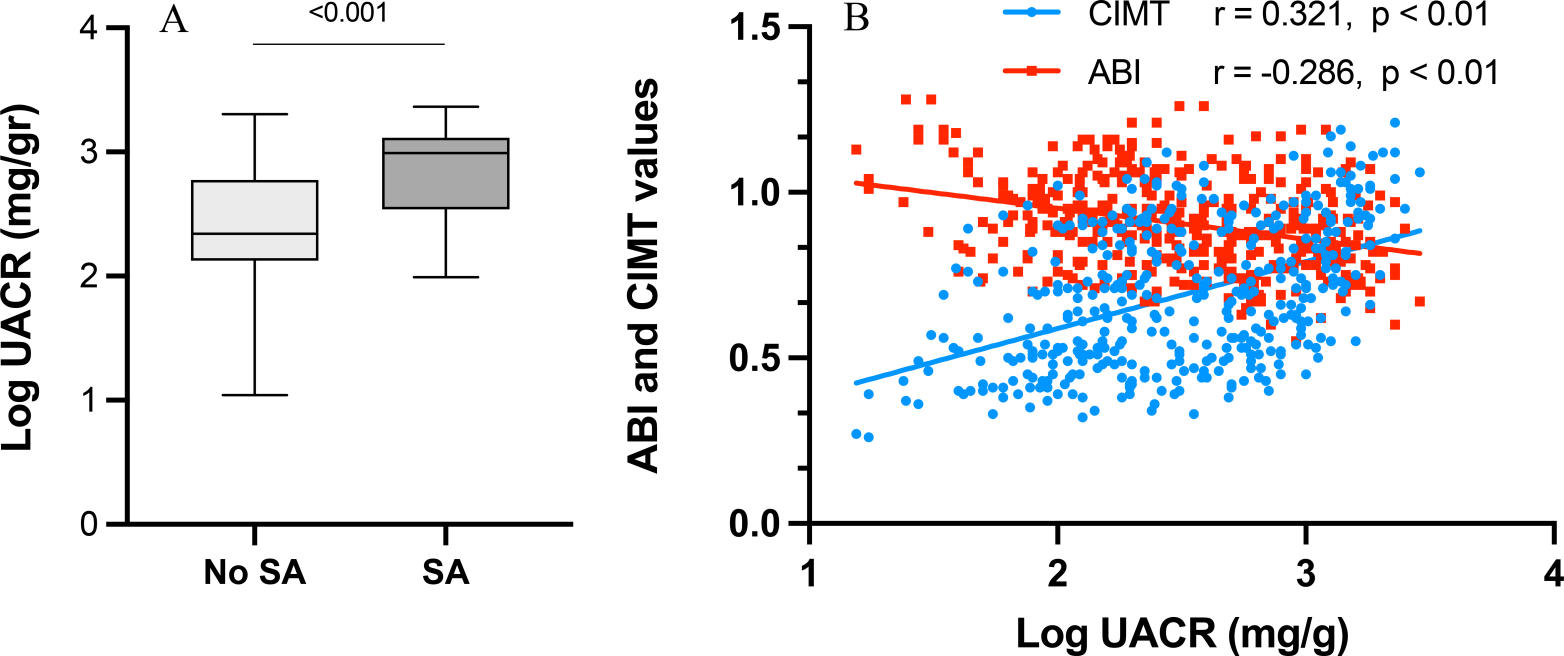
**(A)** Log-transformed UACR values according to the existence of subclinical atherosclerosis (SA). **(B)** Correlations between serum log-transformed UACR levels with ABI and CIMT values. Boxes show median and interquartile range and whiskers indicate the full data range. N = 362.

UACR (log-transformed) demonstrated significant correlations with both vascular measures and inflammatory markers. Higher UACR correlated directly with CIMT (Spearman r = 0.321, p < 0.001) and inversely with ABI (r = –0.286, p < 0.01), consistent with a link between albuminuria and subclinical vascular disease (**Figure 2** and **Table 3**). Log UACR also correlated positively with serum concentrations of IL6 (r = 0.459) and IL1β (r = 0.513, p < 0.001 for both), as well as with SII (r = 0.212, p = 0.035). In peripheral blood leukocytes, UACR was associated with higher mRNA expression of *TNF* (r = 0.256, p = 0.015), *CD36* (r = 0.336, p < 0.001), and *TLR2* (r = 0.361, p = 0.021).

**Table 3:** Bivariate correlations of UACR, CIMT and ABI.

Variable	UACR (Log mg/g)		ABI		CIMT (mm)	
	r	*p*	r	*p*	r	*p*
ABI	-0.286	<0.01				
CIMT (mm)	0.321	<0.01	-0.401	<0.001		
HbA1c (%)	0.378	0.016	0.039	0.809	-0.146	0.37
eGFR (ml/min/1.73 m^2^)	-0.533	<0.001	0.098	0.327	-0.169	0.09
Glucose (mg/dL)	0.34	<0.001	-0.207	0.037	0.19	0.046
IL6 (Log pg/mL)	0.459	<0.001	-0.592	<0.001	0.58	<0.001
IL1β (Log pg/mL)	0.513	<0.001	-0.342	<0.01	0.219	<0.01
SII (x10^9^/L)	0.212	0.035	-0.154	0.143	0.272	<0.01
*TNF* mRNA (Log a.u.)	0.256	0.015	-0.222	0.025	-0.015	0.88
*IL6* mRNA (Log a.u.)	0.147	0.14	-0.023	0.822	0.189	0.047
*CD36* mRNA (Log a.u.)	0.336	<0.001	-0.119	0.031	0.291	0.024
*TLR2* mRNA (Log a.u.)	0.361	0.021	0.049	0.628	0.061	0.251

UACR, urine albumin-to-creatinine ratio; ABI, ankle-brachial index; CIMT, carotid intima-media thickness; HbA1c, hemoglobin A1c; eGFR, estimated glomerular filtration rate; IL, interleukin; SII, systemic immune-inflammation index; TNF, tumor necrosis factor alpha; CD36, cluster differentiation 36; TLR, toll-like receptor; a.u., arbitrary units.

CIMT was positively correlated with serum IL6 (r = 0.58, p < 0.001) and IL1β (r = 0.219, p < 0.01), with SII (r = 0.272, p < 0.01), and with *IL6* and *CD36* circulating leukocyte mRNA expression (r=0.189 and r=0.2091, respectively; p<0.05 for both). Conversely, ABI values showed inverse correlations with fasting glucose (r = –0.207, p = 0.037), serum IL6 (r = –0.592, p < 0.001) and IL1β (r = –0.342, p < 0.01), and leukocyte *TNF* and *CD36* expression (r = –0.222, r = –0.119, respectively; p < 0.05 for both).

### Multivariable analysis

In unadjusted analysis, higher UACR was associated with greater odds of SA (OR per log-UACR 1.84, 95% CI 1.36–2.49, p < 0.001). This association remained significant after adjusting for traditional risk factors, with log-UACR independently associated with presence of SA (adjusted OR 1.72, 95% CI 1.21–2.44, p = 0.002). Among other covariates, age (OR 1.05 per year, p = 0.01) and smoking (OR 1.89, p = 0.03) were significant predictors of SA, whereas sex, BMI, blood pressure, and LDL cholesterol were not significant in the adjusted model.

Sequential logistic regression models were employed to assess the association between SA and inflammatory variables that showed significant or borderline significance in the bivariate correlation analyses (**Table 4**). After adjusting for covariates, increased serum and mRNA levels of IL6 (OR 1.321, 95%CI 1.226-1.923, p = 0.012; and OR 1.502, 95%CI 1.114-2.075, p < 0.01, respectively) together with serum IL1β (OR 1.289, 95%CI 1.172-2.291, p = 0.028) and *TLR2* mRNA levels (OR 1.753, 95%CI 1.201-2.276, p = 0.031) were associated with a higher prevalence of SA. Although leukocyte CD36 expression was higher in patients with SA and in those with macroalbuminuria, this association was attenuated and no longer statistically significant in fully adjusted models.

**Table 4:** Adjusted OR (95% CI) for subclinical atherosclerosis according to inflammatory parameters.

Variable	Model 1		Model 2		Model 3	
	OR (95%CI)	*p*	OR (95%CI)	*p*	OR (95%CI)	*p*
IL6 (Log pg/mL)	1.521 (1.121-1.982)	<0.01	1.439 (1.111-1.865)	<0.01	1.321 (1.226-1.923)	0.012
IL1β (Log pg/mL)	1.981 (1.452-2.159)	<0.01	1.881 (1.512-2.011)	<0.01	1.289 (1.172-2.291)	0.028
SII (Log x10^9^/L)	1.163 (1.003-1.298)	<0.05	1.231 (0.903-1.381)	0.343	1.382 (0.803-1.401)	0.173
*TNF* mRNA (Log a.u.)	1.035 (0.672-2.113)	0.312	0.965 (0.712-2.007)	0.312	0.853 (0.598-2.154)	0.312
*IL6* mRNA (Log a.u.)	1.672 (1.286-2.211)	<0.01	1.743 (1.169-2.349)	<0.01	1.502 (1.114-2.075)	<0.01
*CD36* mRNA (Log a.u.)	1.432 (0.785-2.548)	0.11	1.388 (0.985-2.129)	0.08	1.176 (0.982-2.311)	0.09
*TLR2* mRNA (Log a.u.)	1.834 (1.327-2.786)	0.024	1.769 (1.336-2.698)	0.029	1.753 (1.201-2.276)	0.031

Model 1: crude; Model 2: adjusted for age, sex, and BMI; Model 3: adjusted for age, sex, BMI, UACR, eGFR, smoking status, and hypertension. IL, interleukin; SII, systemic immune-inflammation index; *TNF*, tumor necrosis factor alpha; *CD36*, cluster differentiation 36; *TLR2*, toll-like receptor 2; OR, odds ratio; a.u., arbitrary units.

### Mediation analysis of inflammation in the relationship between UACR and SA

**Figure 3:**
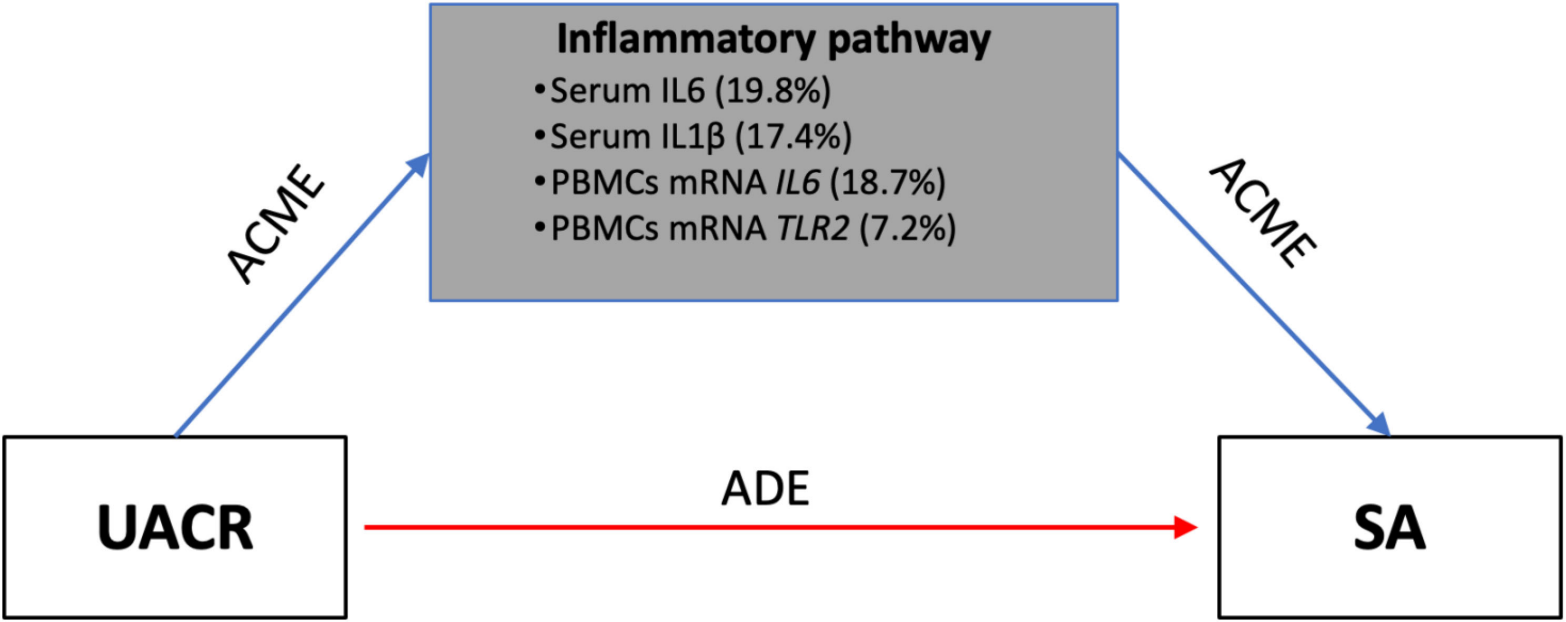
Simplified mediation model of the effect of UACR on subclinical atherosclerosis, illustrating both direct and indirect effects through a composite inflammatory pathway. The pathway aggregates four key mediators: serum IL-6, serum IL-1β, blood leukocyte mRNA IL6, and blood leukocyte mRNA TLR2. Percentages indicate the proportion of the total effect mediated by each marker. ADE, average direct effect; ACME, average causal mediation effect; IL, interleukin; TLR2, Toll-like receptor 2.

We next evaluated whether inflammatory markers mediate the effect of UACR on SA. As presented in **Table 5** and **Figure 3**, serum levels of IL6 and IL1 β and mRNA expression of *IL6* and *TLR2* showed statistically significant indirect effects, indicating partial mediation of the relationship between UACR and SA. The strongest mediating role was observed for serum IL6 (0.024, 95% CI 0.012-0.065, p = 0.004) and leukocyte mRNA *IL6* expression (0.021, 95% CI 0.009-0.063, p = 0.006), which mediated 19.8% and 18.7% of the total effect, respectively. Serum IL1β and leukocyte mRNA *TLR2* also exhibited significant mediation effects, mediating 17.4% and 7.2% of the total effect, respectively. CD36 did not emerge as a significant mediator of the association between albuminuria and SA, and no indirect effect remained significant after Benjamini-Hochberg correction. In all models, the direct effect of UACR on SA remained significant, suggesting that albuminuria is associated with SA both directly and indirectly via inflammatory pathways. The total effect of UACR on SA remained robust and statistically significant across all mediation models (range: 0.108–0.114; all p < 0.01), with bootstrapped 95% confidence intervals excluding the null.

**Table 5:** Mediation of inflammatory markers for the associations between UACR and subclinical atherosclerosis.

Mediator	Indirect effect		Direct effect		Total effect	Proportion mediated
Variable	Coefficient (95% CI)	*p*	Coefficient (95% CI)	*p*	Coefficient (95% CI)	% (95% CI)
IL6 (pg/mL)	0.024 (0.012, 0.065)	0.004	0.070 (0.021, 0.142)	0.010	0.094 (0.062, 0.174)	19.8 (12.1, 42.6)
IL1β (pg/mL)	0.012 (0.005, 0.053)	0.018	0.071 (0.030, 0.146)	0.006	0.098 (0.061, 0.169)	17.4 (8.2, 36.5)
*IL6* mRNA (a.u.)	0.021 (0.009, 0.063)	0.006	0.067 (0.020, 0.136)	0.011	0.108 (0.057, 0.168)	18.7 (15.4, 41.8)
*TLR2* mRNA (a.u.)	0.01 (0.002, 0.041)	0.032	0.012 (0.002, 0.051)	0.004	0.051 (0.024, 0.169)	7.2 (6.3, 28.1)

All models adjusted for age, sex, BMI, UACR, eGFR, smoking status, and hypertension. IL, interleukin; *TLR2*, toll-like receptor 2. Log transformed values of mediators.

## Discussion

In this study, we investigated the mediating role of inflammatory markers—both circulating proteins and circulating leukocyte gene expression—in the relationship between albuminuria and SA in patients with T2DM and moderate CKD. Our findings support the hypothesis that UACR is not merely a marker of glomerular injury but actively contributes to a pro-inflammatory state with systemic vascular consequences.

Previous longitudinal studies have identified albuminuria and reduced eGFR as independent risk factors for cardiovascular events in individuals with diabetes (24). The increased cardiovascular risk in CKD may be partially explained by endothelial dysfunction and accelerated large-vessel atherosclerosis. Consistent with previous research (25, 26), we observed significant associations between UACR and both CIMT and ABI, indicating that even in the absence of overt cardiovascular disease, higher albuminuria correlates with early vascular alterations. The direct association between UACR and CIMT observed in our study, alongside the inverse association with ABI, supports the notion that albuminuria reflects early vascular injury that may precede more advanced atherosclerotic disease in this population. Importantly, our data suggest that elevated UACR is linked to SA independently of traditional risk factors, reinforcing the importance of albuminuria monitoring in diabetic CKD patients as part of cardiovascular risk assessment.

A central finding of our study is the association between elevated UACR and systemic inflammation. Notably, serum IL6 and leukocyte *IL6* mRNA accounted for roughly 40% of the association between albuminuria and SA, underscoring IL6 as a pivotal inflammatory mediator of vascular injury linked to albuminuria. This is consistent with the established pro-atherogenic role of IL6, which includes stimulating hepatic CRP production, endothelial activation, and leukocyte recruitment, key events in vascular injury and plaque formation (26–29). This aligns with the broader literature establishing IL6 as both a marker and driver of cardiovascular risk. Indeed, recent evidence from the MESA cohort indicates that IL6 levels are an even stronger predictor of cardiovascular events than hs-CRP (30), emphasizing its central pathogenic role. Moreover, a recent Phase 2 trial of IL6 inhibition in CKD patients with elevated inflammation (the RESCUE trial) demonstrated that antagonizing IL6 signaling (using the monoclonal antibody ziltivekimab) can dramatically reduce systemic inflammatory biomarkers (31). Similarly, IL1β contributed significantly as a mediator between UACR and SA. IL1β is a major downstream effector of the NLRP3 inflammasome, whose upregulation in CKD has been linked to renal and vascular inflammation, plaque destabilization and atherogenesis. In our analysis, IL1β explained 17.4% of the effects of albuminuria on SA. Notably, targeting of IL1β with canakinumab has shown cardiovascular benefit in individuals with elevated CRP (as evidenced by the CANTOS trial) (32). It is also worth noting that residual inflammatory risk related to upstream mediators such as IL6 persists even after IL1β inhibition (33), highlighting the need to target multiple points in the inflammatory cascade. In any case, RESCUE and CANTOS trials underscore the causal role of inflammation in atherosclerosis and suggest potential therapeutic avenues for high-risk groups including those with DKD. Our findings solidify the concept that specific innate immune cytokines like IL6 and IL1β are not just markers but active drivers of cardiovascular risk in CKD population.

Mediation analysis also revealed a significant, though smaller, mediating effect of *TLR2* gene expression in leukocytes, accounting for approximately 7.2% of the association between UACR and SA. LR2 is an innate immune receptor that recognizes endogenous damage-associated molecular patterns (DAMPs) released during tissue injury. In the context of DKD, excessive filtered albumin can act as a DAMP that activates tubular cells and monocytes via TLRs. TLR engagement triggers NF-κB signaling and induces transcription of cytokines like IL6 and IL1β (34, 35). The upregulation of TLR2 in our patients with higher albuminuria and SA suggests that albuminuria-induced renal inflammation may initiate systemic immune activation through TLR pathways. This is consistent with experimental data showing that TLR activation in the kidney exacerbates inflammation and injury (36). Our findings thus support a model wherein albuminuria is associated with TLR signaling, and may contribute to systemic vascular inflammation. Notably, increased expression of TLR2 and TLR4 – receptors known to mediate responses to DAMPs – may further amplify the inflammatory burden in patients with DKD and SA (37).

Our findings align with previous studies demonstrating a strong correlation between albuminuria and systemic inflammation and support the role of albuminuria as a biological amplifier of systemic inflammation, contributing to vascular remodeling and early atherosclerosis (38–40). Albuminuria appears to function as more than a passive marker, instead acting as a biological amplifier of inflammation that promotes vascular remodeling. The mechanistic basis for this likely involves multiple interrelated pathways. Excessive filtered albumin acts as a DAMP, activating inflammatory pathways such as the NLRP3 inflammasome in tubular epithelial cells, and stimulating the release of cytokines that can enter systemic circulation, contributing to low-grade systemic inflammation and vascular dysfunction (27, 40, 41). Moreover, tubular epithelial cells exposed to albumin release extracellular vesicles, including exosomes carrying mRNA and protein cargo such as CCL2. These can be taken up by immune cells, including macrophages, enhancing their activation and promoting renal inflammation (42). Our results are in accordance with this mechanism supporting for the hypothesis that systemic inflammation through both humoral (cytokine) and cellular (gene expression) pathways mediates the contribution of albuminuria to early atherogenesis. The graded mediation effects for IL6, IL1β, and TLR2 suggest the involvement of multiple branches of the inflammatory cascade – humoral (cytokine-mediated), cellular (leukocytes activation), and innate immune receptor pathways – likely interacting synergistically.

A novel aspect of our study is the integrated analysis of both serum cytokine levels and gene expression in peripheral blood leukocytes. Changes in peripheral leukocyte gene expression reflect systemic immune alterations and have been implicated in the pathogenesis of both DKD and atherosclerosis (43–48). Altered mRNA expression in peripheral leukocytes—including lymphocytes and mononuclear cells—has been reported in patients with diabetes, diabetic nephropathy, CKD, metabolic syndrome, and advanced atherosclerosis (19, 20, 49, 50). Thus, in patients with carotid atherosclerosis, pro-inflammatory gene signatures in circulating monocytes mirror those found in plaque-resident immune cells. Our finding that leukocyte expression of genes like *TNF*, *TLR2*, and *CD36* correlates with albuminuria and SA supports the concept of a systemic “inflammatory fingerprint” linking kidney and vascular pathology. Notably, Sternberg et al. demonstrated that immune cells isolated from atherosclerotic plaques and peripheral blood share common inflammatory activation patterns, underscoring the interplay between circulating mononuclear cells and vascular inflammation (21). By concurrently assessing soluble biomarkers and circulating leukocyte mRNA, our study provides a more comprehensive view of the inflammatory state in DKD and its contribution to atherogenesis.

CD36 is mechanistically linked to oxidized LDL uptake, foam-cell formation, and early atherogenesis, which makes it a biologically plausible marker. However, in our dataset, although leukocyte CD36 expression was higher in individuals with SA and macroalbuminuria, its association with SA was attenuated and no longer significant after multivariable adjustment. CD36 also did not emerge as a significant mediator in our exploratory mediation analyses, particularly after FDR correction. These findings suggest that its independent contribution beyond IL6, IL1β, and TLR2 is limited, possibly reflecting collinearity with related innate immune pathways or insufficient power to detect a modest effect. Therefore, no firm mechanistic conclusions can be drawn regarding CD36 in this cohort, and its role warrants further investigation.

Beyond IL6 and IL1β, other cytokines such as IL-18 have also been implicated in atherosclerosis and vascular inflammation and may represent additional mechanistic links between metabolic disease and vascular injury, although these pathways were not directly assessed in the present study (51). In addition to kidney-derived inflammatory signals, obesity- and NAFLD-associated inflammation represents an important upstream contributor to systemic inflammatory burden in T2DM. Hepatic steatosis and visceral adipose tissue dysfunction have been associated with increased carotid intima-media thickness and with cytokines such as IL-18, IL-15, and IL-17 in prior studies, suggesting that vascular injury in diabetes reflects the convergence of multiple metabolic inflammatory pathways rather than a single organ-specific mechanism (14, 51–53).

It is important to note that several observed correlations, although statistically significant, were of modest magnitude. Such small effect sizes suggest limited standalone biological impact and highlight that inflammation likely acts through multiple overlapping pathways rather than a single dominant mediator. Accordingly, these correlations should be interpreted cautiously and not overstated.

While our findings are compelling, several limitations warrant consideration. The pattern of associations suggests that inflammation may help explain why higher albuminuria coincides with a greater vascular disease burden. However, because the study is cross-sectional, these observations are not evidence of causality and we cannot definitively prove that albuminuria causes inflammation-mediated atherosclerosis, only that they are strongly associated. Thus, all associations reported here should be interpreted as non-causal and hypothesis-generating. Prospective studies are needed to confirm temporal relationships and causality. Second, unmeasured confounders may be present despite multivariable adjustment; for instance, we did not have data on dietary factors or gut microbiota-derived toxins that might influence inflammation. Third, this was a single-center study involving a relatively homogenous population in which all participants received uniform background therapy with RAS blockers and statins, which may limit generalizability of our findings to patients not receiving these treatments or to broader ethnic or geographic groups. Fourth, SA was assessed by CIMT and ABI rather than direct imaging of plaques; although these are established markers, they are surrogate measures of atherosclerosis. Mediation analysis assumptions should be acknowledged – we modeled the mediating effect of each inflammatory marker independently, although these pathways overlap and may interact. Fifth, our analysis focused on select inflammatory pathways; other mediators such as oxidative stress, adipokines, or components of adaptive immunity, as well as residual confounding factors like homocysteine, were not measured and may also contribute to the albuminuria–CVD link. In addition, other unmeasured clinical or lifestyle factors may also have influenced the inflammatory and vascular profiles observed. Finally, although we excluded patients with acute illness or recent nephrotoxic drug exposure, some residual short-term variability in UACR is possible and may have attenuated certain associations. Moreover, as the study was conducted before the introduction of SGLT2 inhibitors and finerenone, our findings reflect a pre–SGLT2/finerenone therapeutic era. Despite these limitations, our study has notable strengths. To our knowledge, this is one of the first studies to integrate circulating cytokine profiling with circulating leukocytetranscriptomic data in DKD patients to elucidate links with atherosclerosis. All patients were on uniform background therapies (RAS blockade and statins), which may reduce confounding by treatment differences. We rigorously adjusted for key clinical covariates and employed bootstrapped mediation analysis to better quantify indirect effects. The consistency of findings across serum and cellular markers (e.g. serum and leukocyte mRNA IL-6 levels) strengthens the biological plausibility of our conclusions.

In conclusion, this study provides new insights into how albuminuria is associated with systemic inflammation and early vascular injury in patients with DKD. We demonstrate that elevated albuminuria is associated with a heightened inflammatory state – characterized by increased IL6, IL1β, and innate immune receptor expression – which in turn contributes to the development of SA. Our findings support the concept of the kidney as a source and target of inflammatory signaling in DKD, potentially contributing to systemic inflammatory activation. Identifying IL6 and TLR2 as key mediators suggests potential therapeutic avenues. For example, interventions that reduce albuminuria (such as SGLT2 inhibitors or optimized RAS blockade) or that target inflammation (e.g. IL1β or IL6 inhibitors, or novel TLR antagonists) might attenuate the propagating cycle of renal and vascular injury (32). Indeed, broad anti-inflammatory approaches like low-dose colchicine have recently shown efficacy in reducing cardiovascular events in non-CKD populations (32), highlighting inflammation as a tractable target. Future studies should explore whether anti-inflammatory therapies can specifically improve outcomes in patients with DKD and whether biomarkers such as IL6 or TLR2 could help stratify cardiovascular risk in CKD. As the prevalence of diabetes and CKD continues to rise, a deeper understanding of the kidney–heart connection will be essential for developing strategies to prevent cardiovascular complications in this high-risk group.

## Data availability statement

The datasets presented in this study can be found in online repositories. The names of the repository/repositories and accession number(s) can be found in the article/**Supplementary Material**.
