# Supplementary material for: Inflammatory markers associated with albuminuria and early atherosclerosis in type 2 diabetic kidney disease: a cross-sectional study
Source: Front Endocrinol (Lausanne). 2026 Feb 26;17:1732900. doi: 10.3389/fendo.2026.1732900 (PMC12979170; doi:10.3389/fendo.2026.1732900)
Supplement: Supplementary file 1 [file DataSheet1.docx]

**Supplementary Table 1**. TaqMan Assays IDs and accession numbers for the corresponding reference sequences of each mRNA.

| Target gene | Assay ID | Accession number | Amplicon length (bp) |
| --- | --- | --- | --- |
| *TNF* | Hs00174128 m1 | NM_000594 | 80 |
| *IL1β* | Hs00174097_m1 | NM_000576 | 94 |
| *IL6* | Hs00985639_ml | NM_000600 | 66 |
| *IL10* | Hs00961622_m1 | NM_000572 | 74 |
| *CD36* | Hs01567185_g1 | NM_000072 | 116 |
| *CCL2* | Hs00234140_m1 | NM_002982 | 101 |
| *TLR2* | Hs00152932_m1 | NM_003264 | 80 |
| *TLR4* | Hs00152939_m1 | NM_003266 | 89 |
| *NFkB* | Hs00765730_m1 | NM_003998 | 66 |
| *GAPDH* | Hs99999905_m1 | NM_001256799 | 122 |

TNF, tumor necrosis factor alpha; IL1β, interleukin 1β; IL6, interleukin 6; IL10, interleukin 10; CD36, cluster differentiation 36; CCL2, C-C motif chemokine ligand 2; TLR, toll-like receptor; NFkB, nuclear factor-kappa B; GAPDH, glyceraldehyde-3-phosphate dehydrogenase.
